# *In Vitro* Bioactivity of Methanolic Extracts from* Amphipterygium adstringens* (Schltdl.) Schiede ex Standl.,* Chenopodium ambrosioides* L.,* Cirsium mexicanum* DC.,* Eryngium carlinae* F. Delaroche, and* Pithecellobium dulce* (Roxb.) Benth. Used in Traditional Medicine in Mexico

**DOI:** 10.1155/2018/3610364

**Published:** 2018-02-28

**Authors:** Peter Knauth, Gustavo J. Acevedo-Hernández, M. Eduardo Cano, Melesio Gutiérrez-Lomelí, Zaira López

**Affiliations:** ^1^Cell Biology Laboratory, Centro Universitario de la Ciénega, Universidad de Guadalajara, Av. Universidad 1115, 47810 Ocotlán, JAL, Mexico; ^2^Laboratorio de Biología Molecular Vegetal, Centro Universitario de la Ciénega, Universidad de Guadalajara, Av. Universidad 1115, 47810 Ocotlán, JAL, Mexico; ^3^Laboratorio de Biofísica, Centro Universitario de la Ciénega, Universidad de Guadalajara, Av. Universidad 1115, 47810 Ocotlán, JAL, Mexico; ^4^Laboratorio de Alimentos, Centro Universitario de la Ciénega, Universidad de Guadalajara, Av. Universidad 1115, 47810 Ocotlán, JAL, Mexico

## Abstract

Seven out of eight methanolic extracts from five plants native to Mexico were inactive against ten bacterial strains of clinical interest. The fruit extract of* Chenopodium ambrosioides* inhibited the bacteria* Enterococcus faecalis* (MIC = 4375 *μ*g/ml),* Escherichia coli* (MIC = 1094 *μ*g/ml), and* Salmonella typhimurium* (MIC = 137 *μ*g/ml). The fruit extract of* C. ambrosioides* was with CC_50_ = 45 *μ*g/ml most cytotoxic against the cell-line Caco-2, followed by the leaf extract from* Pithecellobium dulce* (CC_50_ = 126 *μ*g/ml); interestingly, leaves of* C. ambrosioides* (CC_50_ = 563 *μ*g/ml) and bark of* P. dulce* (CC_50_ = 347 *μ*g/ml) extracts were much less cytotoxic. We describe for the first time the cytotoxic effect from extracts of the aerial parts and the flowers of* Cirsium mexicanum* (CC_50_ = 323 *μ*g/ml and CC_50_ = 250 *μ*g/ml, resp.). Phytochemical analysis demonstrated for both extracts high tannin and saponin and low flavonoid content, while terpenoids were found in the flowers. For the first time we report a cytotoxicological study on an extract of* Eryngium carlinae* (CC_50_ = 356 *μ*g/ml) and likewise the bark extract from* Amphipterygium adstringens* (CC_50_ = 342 *μ*g/ml). In conclusion the fruit extract of* C. ambrosioides* is a potential candidate for further biological studies.

## 1. Introduction

Plants are well known as a rich source of bioactive compounds with a wide range of pharmaceutical applications, for instance, neuro- or psychoactive compounds (e.g., atropine, caffeine, cocaine, and morphine), substances with anti-inflammatory (e.g., acetylsalicylate) or cardiovascular effects (e.g., digoxin), or anticancer activity (e.g., taxol, vinblastine). Hence, over the last few decades raw extracts from medicinal plants have been studied in order to discover pure compounds that show any of the aforementioned biological activities.

Most of the antibiotics used in clinical and veterinary medicine are from microbial origin (bacteria, fungi) and only a few of them are synthetic. However, since the end of the “Golden Age of Antibiotic Discovery” in the 80s of the last century only a small number of new antibiotics have emerged, while on the other hand more and more antibiotics have lost activity due to the rise of acquired resistance against them [1]. In 2005, the World Health Organization (WHO) developed a list of Critically Important Antimicrobials (CIA) for human medicine and published a global action plan to address this problem [2]. Therefore, now other niches (e.g., marine environment) and sources (e.g., animals or plants) are being investigated to discover new classes of antibiotics [3]. Mexico possesses a very high plant biodiversity with about 26,000 species already identified [4], and an estimated 4,500 plants are used for medicinal purposes [5]; but* in vitro* antibacterial properties have been reported for only 343 plant species [6]. Although many phytochemicals (e.g., eugenol, menthol, thymol, and linalool) or plant peptides with antimicrobial activity are known, those molecules are normally not used as antibiotics. Moreover, among those medicinal plants from Mexico, only 300 of them have documented use in anticancer treatment: 181 are supported by scientific evidence and 119 have been used empirically [7].

*Amphipterygium adstringens* (Schltdl.) Schiede ex Standl., commonly known in Mexico as cuachalalate, is widely used as a medicinal plant, especially to treat ulcers, cancer, gastritis, and other gastrointestinal disorders as well as promote wound healing [8]. Extracts from the bark of this plant have antibacterial activity, which is attributed to a mixture of anacardic acids (Table 1). At a concentration of 10–40 *μ*M those anacardic acids were cytotoxic to several cell lines, while other authors reported proliferative effects (Table 1). Moreover, extracts containing masticadienonic acid and its 3*α*-hydroxy-derivative exhibited in rats antiulcer activity [9] and anti-inflammatory effects [10].

*Chenopodium ambrosioides* L., also known as “wormseed,” “Jesuit's tea,” or “epazote,” has been empirically used in Mexico as an infusion for its antiparasitic activity and other positive effects on the gastrointestinal tract [11]: among its components, especially ascaridole and hydroperoxide derivates from monoterpenes with two double bonds exhibit antiprotozoal activity (Table 1). Moreover, its essential oils have antifungal activity; and methanol or petroleum ether extracts of the bark or flowers have antibacterial effects (Table 1). There are, however, contradictory reports on cytotoxic effects of its extracts (Table 1). Additionally, moderate antioxidant and anti-inflammatory properties could be attributed to the chenopodiumamines A to C isolated from ethanolic extracts [12]. Using an animal model, ethanolic extracts of leaves or stems exhibited anti-inflammatory, antinociceptive, and healing effects, which validate its potential therapeutical use [13].

*Cirsium mexicanum* DC., known as “Mexican thistle” or “cardosanto,” is empirically employed by the Mexican population to treat cancer or diabetes [14], but there is no scientific contribution about the biological activity of this plant.

*Eryngium carlinae* F. Delaroche, known in Mexico as “hierba del sapo,” is often used in traditional medicine to treat diabetes. Although an ethanolic extract of the whole plant of* E. carlinae* did not lower blood glucose levels in diabetic rats, a significant decrease of creatinine, urate, total cholesterol, and triglycerides could be demonstrated, which may lower the risk of renal and cardiovascular complications caused by diabetes mellitus [15]. Additionally, a methanolic extract from* E. carlinae* inhibits growth of* Helicobacter pylori* [16].

Finally,* Pithecellobium dulce* (Roxb.) Benth. is autochthonous to Mexico and Central America, where it is known as “guamúchil” or “huamúchil,” but nowadays it can also be found in Southeast Asia and India, where it is known as “Madras thorn.” In Mexico, the plant is traditionally prepared as aqueous infusions or ethanolic extracts from different parts of the tree: from leaves to alleviate pain and convulsions and combined with salt to cure indigestion and from bark for treatment of dysentery or fever [17]. In rats, an ethanolic seed extract has protective effects against ulceration [18] and antidiabetic activity [19], while an aqueous leaf extract shows antihyperlipidemic activity [20]. A hydroethanolic extract of the bark exhibited antimicrobial activity (Table 1). Recent cytotoxicological studies revealed cytotoxic effects of a methanolic leaf extract on the prostate cancer cell line CaP, while no effect on the breast cancer cell line MCF-7 was observed [21].

When plants are selected on the basis of their use in traditional medicine, the first extraction step to obtain a raw extract should mimic the extraction process as described by the traditional healer [22]. In an initial screening for biological activity, the concentration ranges usually from 10 to 10,000 *μ*g/ml, but only raw extracts with a half maximal effective concentration EC_50_ ≤ 100 *μ*g/ml are considered active [23] and subjected to further analysis.

Of the five plant species included in the present study, two have not been extensively studied scientifically and for the remaining three plants the published literature shows contradictory results concerning their cytotoxicity. Therefore, we considered these five plants, which are all endemic to Mexico, as good candidates for assessment of their* in vitro* cytotoxicity using the human epithelial colorectal adenocarcinoma cell line Caco-2, a standard model to discover novel anticancer agents in colon cancer. Additionally, their antimicrobial activity, useful to discover novel agents to prevent and treat nosocomial infections, was evaluated.

## 2. Methodology

### 2.1. Plant Material and Extraction

Dried bark of* A. adstringens* was bought at a local market in Ocotlán (Jalisco, Mexico), while the samples of* C. ambrosioides*,* C. mexicanum*,* E. carlinae,* and* P. dulce* were collected in “La Ciénega" region (Jalisco, Mexico) between June and July. All fresh material was delivered to the laboratory and dried at room temperature (RT) by ventilation for 10 d. Afterwards the collected material was separated according to plant parts (Table 1). Each part was ground (electrical grinder) and passed through a 30 mesh sieve to get a fine powder. Then 10 g of plant powder from each sample was macerated with 100 ml methanol by shaking at 80 rpm for 24 h at RT. Next, the macerates were filtered, first through Whatman filter paper #4 and then through a Nylon membrane 0.45 *μ*m pore size. Finally, 4 ml of the filtrates was distributed into amber vials, the solvent was evaporated using a nitrogen evaporator (Mini-Vap, CRS, Louisville, USA), the vial was weighed, and the precipitate was resuspended in 1 ml methanol. All samples were stored at 4°C until use.

### 2.2. Molecular Identification through DNA Barcoding

DNA barcoding is a molecular method which allows the rapid, accurate, and automatic identification of plants using DNA sequences. This method has been tested in numerous plant species and has been proposed as an alternative for the authentication of medicinal plants [35, 36]. First, plant powder was obtained as described above, and 100 mg from each sample was used for DNA isolation using the PureLink Plant Total DNA Purification Kit (Invitrogen, Carlsbad, USA), following the protocol suggested by the manufacturer. To test the quality of isolated DNA, a 5 *μ*l sample was run on a 0.8% agarose gel stained with EvaGreen (Jena Bioscience, Jena, Germany). Quantification of DNA was achieved by UV spectrophotometry using a NanoDrop 2000 Spectrophotometer (Thermo Fisher Scientific, Waltham, USA). Primers and PCR conditions were as those reported by the Consortium for Barcode of Life (CBOL) Plant Working Group [35]. Primers used for amplification of* matK* gene were 3F-KIM-F (5′-CGT ACA GTA CTT TTG TGT TTA CGA G-3′) and 1R-KIM-R (5′-ACC CAG TCC ATC TGG AAA TCT TGG TTC-3′), and for amplification of* rbcL* gene primers rbcL*a*-F (5′-ATG TCA CCA CAA ACA GAG ACT AAA GC-3′) and rbcL*a*-R (5′-GTA AAA TCA AGT CCA CCR CG-3′) were used. Amplification reactions were performed in a final volume of 20 *μ*l containing 20 mM Tris-HCl (pH 8.4), 50 mM KCl, 2 mM MgCl_2_, 0.2 mM dNTPs mix, 1 *μ*M each primer, 10 ng of genomic DNA and 2 units of Taq DNA polymerase (Jena Bioscience, Jena, Germany). Amplifications were performed in a Multigene Thermocycler (Labnet International, Edison, USA) using the program: 1 step at 94°C for 5 min, 35 cycles consisting of 30 s at 94°C, 1 min at 52°C, and 1 min at 72°C and a final extension step of 5 min at 72°C. Amplification products were separated by electrophoresis in 0.8% agarose gels and stained with EvaGreen. The PCR products were purified using the GenElute PCR Clean-Up Kit (Sigma-Aldrich, St. Louis, USA) following the manufacturer's instructions. The DNA concentration of the samples was again estimated using a NanoDrop Spectrophotometer and PCR products were sent for sequencing to the Genomic Services Laboratory at Langebio-Cinvestav (Irapuato, Mexico). DNA sequences were used for plant identification through the Identification Engine at the Barcode of Life Data System (http://www.boldsystems.org) and were submitted to the public sequence repository GenBank.

### 2.3. Phytochemical Characterization

Only plants or parts of them, which have not been analysed previously, were phythochemically characterized [37]: fine plant powder was suspended at 100 mg/ml in distilled water with shaking (80 rpm) overnight at RT in the dark and subsequently centrifuged (2 min, 10,000 ×g).

*Flavonoids*. 1 ml supernatant, diluted with 900 *μ*l distilled water, was mixed with 5 ml 1% NH_4_Cl and then filtered. Afterwards, 5 drops of concentrated H_2_SO_4_ were added. An unstable yellow colour is formed in the presence of flavonoids.

*Tannins*. 1 ml supernatant was boiled for 2 min and then 5 drops of 0.1% FeCl_3_ were added. A brownish green or blue black colour indicates the presence of tannins.

*Terpenoids*. 100 *μ*l methanolic plant extract, diluted with 5 ml methanol, was mixed with 2 ml dichloromethane and then 3 ml concentrated H_2_SO_4_ was added. After 20 min a stable layer was formed and a reddish-brown colour in the interface indicates positive results for terpenoids.

*Saponins*. 5 ml supernatant was boiled for 2 min, diluted with distilled water, and shaken to form foam; then 3 drops of olive oil were added and again shaken vigorously. The formation of an emulsion indicates the presence of saponins.

### 2.4. Antimicrobial Assays

The following microorganisms were acquired from ATCC:* Bacillus cereus* #49064,* Enterococcus faecalis *#29212,* Klebsiella pneumoniae* #10031,* Salmonella typhimurium* #29630,* Streptococcus pyogenes* #19615 and the following are obtained from the Microbiology Laboratory collection (CUCI, University of Guadalajara):* Escherichia coli*,* Proteus mirabilis*,* Pseudomonas aeruginosa*,* Serratia marcescens,* and* Staphylococcus aureus* [38]. Agar plates with Lysogeny Broth (LB; MP Biomedicals, Santa Ana, USA) or Müller-Hinton (MH; MP Biomedicals, Santa Ana, USA) were inoculated with 10^7^ cfu of the model organism. Sterile sensi-discs (6 mm diameter) were impregnated with 2 *μ*l and 10 *μ*l extract (20–80 mg/ml), 2 *μ*l chloramphenicol (Cm, 25 mg/ml; Sigma-Aldrich, St. Louis, USA) was used as positive control, and 10 *μ*l methanol was used as mock control. After their incubation at 37°C for 24 h the diameter of the inhibition zone was measured (Kirby-Bauer method). For extracts with positive results the minimal inhibitory concentration (MIC) was determined using 96-well microtiter plates (MTP): all wells contained 50 *μ*l medium and were serially diluted starting with 50 *μ*l extract (or control) in the first row; finally all wells were inoculated with 50 *μ*l medium adjusted to 0.5 McFarland (approx. 10^8^ cfu/ml). The MTPs were incubated at 37°C for 24 h and the growth was measured by absorption at 625 nm with an ELISA-reader (Multiskan FC, Thermo Fisher Scientific, Waltham, USA). All tests were done six times.

### 2.5. Cell Line and Culturing Conditions

The cell line Caco-2 (ATCC, Manassas, USA) was grown in DMEM/F12 (Caisson, Smithfield, USA) supplemented with 10% FBS (Biowest, Kansas City, USA) at 37°C, 4% CO_2_, and 95% RH. Experiments were carried out in 96-well MTP containing 100 *μ*l medium inoculated with 10^4^ cells. After 24 h incubation for cell attachment 100 *μ*l of methanolic plant extract (40–60 mg/ml) or control (methanol or H_2_O_2_) was added and serially diluted 1 : 2. Afterwards, the cells were incubated for additional 24 h before the analysis. For qualitative assays 1.5 *∗* 10^5^ cells were grown in 1 ml medium in 12-well MTP on cover slides and otherwise using the same culturing conditions.

### 2.6. Cell Viability and Necrosis Induction

To determine the metabolic activity the old medium was removed, the cells were washed twice with phosphate buffered saline (PBS), and 100 *μ*l medium containing 2 *μ*l WST-1 was added. After incubating for 4 h absorbance at 440 nm was measured with an ELISA-reader (Multiskan FC). Neutral red uptake (NRU) and trypan blue assays were made in 12-well MTP and the cells were grown on cover slides. After the last 24 h of incubation 20 *μ*l of 0.33% neutral red solution (Santa Cruz, Santa Cruz, USA) was added into each well and incubated for additional 4 h. Afterwards, the cover slides were washed once with PBS and observed under the microscope (Axioskop 40FL, Zeiss, Oberkochen, Germany). To detect necrosis, 0.05% (final concentration) trypan blue (Biowest) was added and incubated for 5 to 10 min and the cover slides were washed with PBS and then observed under the microscope (Zeiss Axioskop 40FL). All tests were done in triplicate.

### 2.7. Statistical Analysis

Values are expressed as means ±1.96 *∗* standard error of mean (1.96 *∗* SEM). Differences between groups were determined by one-way analysis of variance (ANOVA) and subjected post hoc to Tukey's HSD multiple comparison tests using the program Origin 5.0 (Microcal Software, Northampton, USA). A value of *p* < 0.05 was considered to indicate statistical significance. The half maximal cytotoxic concentration (CC_50_) was calculated by nonlinear curve fit with dose-response function using the program Origin 5.0.

## 3. Results

### 3.1. Phytochemical Analysis

Methanolic extracts from different parts of* A. adstringens*,* C. ambrosioides*,* C. mexicanum*,* E. carlinae,* and* P. dulce* were prepared at concentrations ranging from 20 to 80 mg/ml and designated by the abbreviations shown in Table 1.

As many reports on the composition of* A. adstringens*,* C. ambrosioides,* and* P. dulce* are already published elsewhere, those were not analysed here. Thus, the phytochemical composition of four extracts that have not yet been characterized was determined qualitatively (Table 2). This analysis revealed that the fruits of* C. ambrosioides* do not contain saponins.

### 3.2. Molecular Identification

All plants collected in the wild were identified through PCR amplification and DNA sequencing of regions of* rbcL* and* matK* plastid genes, which have been proposed as the core plant barcode [35]. For* rbcL* fragments, the DNA sequence length was between 527 and 529 bases, while the sequence length of* matK* was between 807 and 813 bases. For similarity searches, these sequences were submitted to the Barcode of Life Data Systems (http://www.boldsystems.org/) using the* Plant Identification* tool and plant species showing that the top matching scores are depicted in Table 3 along with the corresponding GenBank accession numbers for the DNA sequences obtained in this work. Despite several attempts, the* matK* primers failed to amplify the DNA from* E. carlinae*; thus identification of this plant could only be achieved using* rbcL* sequence. It must be noticed, however, that at the moment neither the* matK* nor the* rbcL* sequences for* E. carlinae *are available at the Barcode of Life Data System, and hence the best matches were closely related species.* A. adstringens* has been identified earlier [38].

### 3.3. Antimicrobial Activity

In the screening for antimicrobial activity by the Kirby-Bauer method only the fruit extract from* C. ambrosioides* caused a clear zone of inhibition against the three strains of* E. faecalis*,* S. typhimurium,* and* E. coli*; as expected, the raw extract ESM had a significant lower activity than a pure antibiotic (Table 4). Hence, only these combinations were subjected to a MIC determination (Table 5). None of the other extracts showed any effect on the tested bacterial strains.

### 3.4. Cytotoxicity

**Figure 1:**
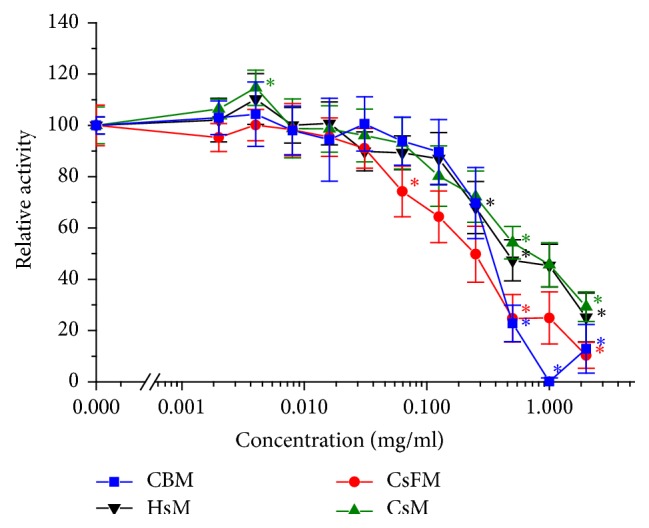
Cytotoxicity of methanolic extracts on the cell line Caco-2: bark of* A. adstringens* (CBM, square), whole plant of* E. carlinae *(HsM, down triangle), and flower (CsFM, circle) and whole plant (CsM, up triangle) of* C. mexicanum*. Error bars indicate 1.96 *∗* SEM (*n* = 6); *∗* indicates statistically significant difference to control and to the next smaller value (*p* < 0.05).

**Figure 2:**
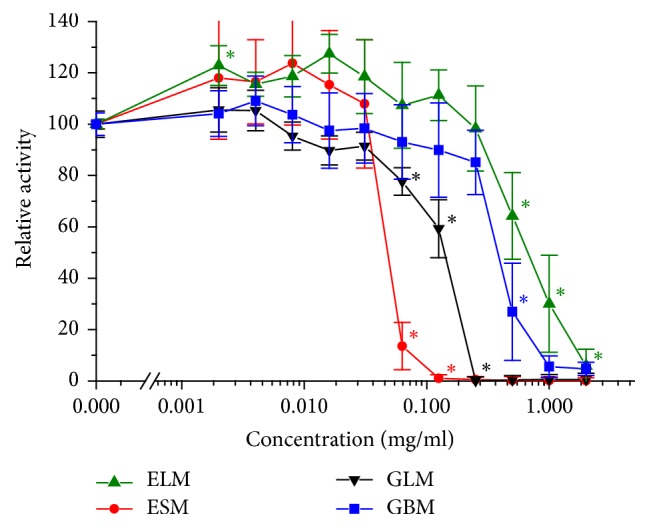
Cytotoxicity of methanolic extracts on the cell line Caco-2: leaf (ELM, up triangle) and fruit (ESM, circle) of* C. ambrosioides* as well as leaf (GLM, down triangle) and bark (GBM, square) of* P. dulce*. Error bars indicate 1.96 *∗* SEM (*n* = 6). *∗* indicates statistically significant difference to control and to the next smaller value (*p* < 0.05).

The cytotoxicity of the extracts on the enterocyte cell line Caco-2 was estimated by the reduction of the tetrazolium salt WST by dehydrogenase activity. Most of the tested methanolic plant extracts had a cytotoxic concentration of CC_50_ ≥ 250 *μ*g/ml, which are considered low cytotoxicity values: the bark of* A. adstringens* (CBM) with CC_50_ = 342 ± 38 *μ*g/ml, flowers (CsFM) and whole plant (CsM) of* C. mexicanum* with CC_50_ = 250 ± 55 *μ*g/ml and with CC_50_ = 323 ± 93 *μ*g/ml, respectively, and the whole plant of* E. carlinae* (HsM) with CC_50_ = 356 ± 114 *μ*g/ml (Figure 1). While for* P. dulce* the bark extract (GBM) was not cytotoxic either with CC_50_ = 347 ± 78 *μ*g/ml, its leaf extract (GLM) exhibited first signs of cytotoxicity with a CC_50_ = 126 ± 14 *μ*g/ml. This contrast between different parts of a plant was even more pronounced for the* C. ambrosioides* extracts: while the leaf extract (ELM) was the least cytotoxic with CC_50_ = 563 ± 66 *μ*g/ml, the fruit extract (ESM) was the most cytotoxic one with CC_50_ = 45 ± 7 *μ*g/ml (Figure 2).

**Figure 3:**
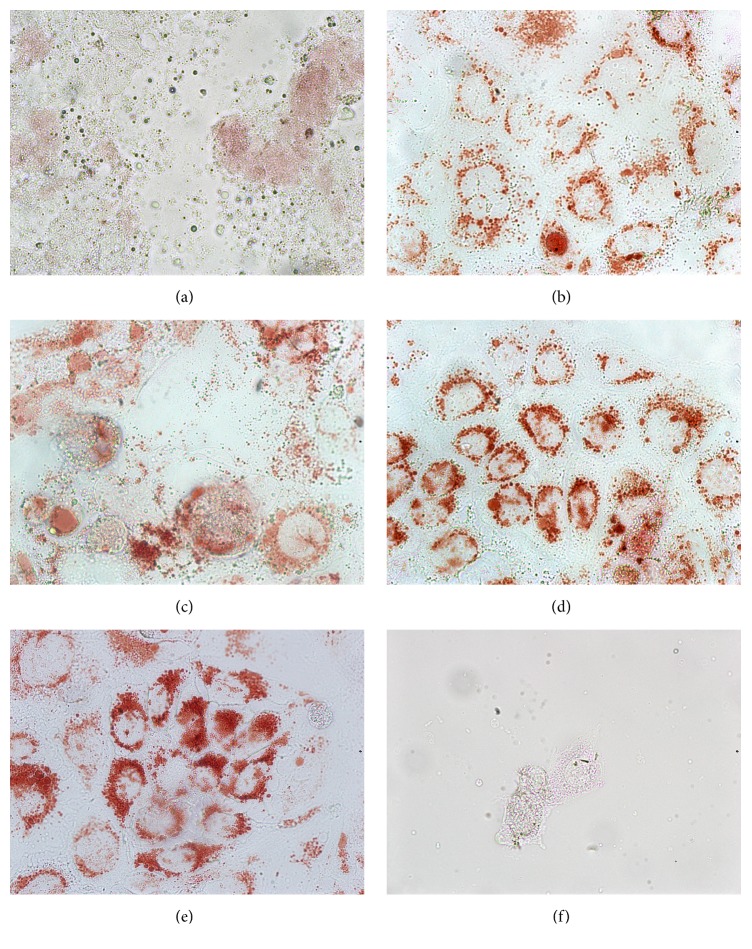
Neutral red uptake of Caco-2 after 24 h exposure to different methanolic extracts: (a) bark of* A. adstringens* (400 *μ*g/ml CBM); (b) whole plant of* C. mexicanum* (400 *μ*g/ml CsM); (c) flowers of* C. mexicanum* (480 *μ*g/ml CsFM); (d) whole plant of* E. carlinae *(500 *μ*g/ml HsM). (e) Methanol as negative control and (f) H_2_O_2_ as positive control.

These results were confirmed qualitatively by neutral red uptake (NRU), where functional lysosomes are stained red and trypan blue staining (TBS), where necrotic cells with disrupted membranes are stained blue. At 400 *μ*g/ml of CBM, Caco-2 cells started to show irregular shapes, although the confluence was not reduced and most cells still had functional lysosomes (i.e., red by NRU, Figure 3(a)) and membranes. At 800 *μ*g/ml of CBM the confluence was clearly reduced and the cells had only a faint red staining by NRU (not shown) and most were blue by TBS (not shown). CsM at 400 *μ*g/ml caused no observable changes in confluence, cell shape, membrane functionality (not shown), and lysosome functionality (Figure 3(b)) and even at a much higher concentration of 2,000 *μ*g/ml of CsM most cells were still stained red by NRU, although the confluence was clearly reduced (not shown). The extract CsFM at 480 *μ*g/ml caused no observable changes in confluence, cell shape, membrane functionality (not shown), and lysosome functionality (Figure 3(c)); but at 1,200 *μ*g/ml of CsFM confluence was clearly reduced and several cells were stained blue by TBS while by NRU the cells were not stained or only slightly red (not shown). The extract HsM at 500 *μ*g/ml did not cause observable cellular alterations; that is, the confluence did not decrease and the cells had functional membranes (not shown) and functional lysosomes (red by NRU, Figure 3(d)), similar to the negative control (Figure 3(e)). At 1,500 *μ*g/ml of HsM the red staining of the cells by NRU was fainter (not shown) but at 3,000 *μ*g/ml of HsM the confluence was clearly reduced and the cells were not stained red by NRU but blue by TBS (both not shown).

**Figure 4:**
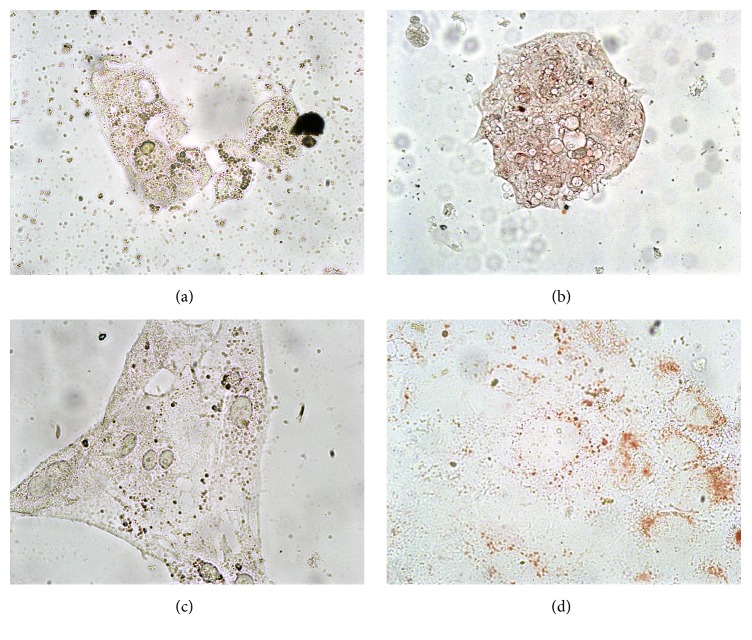
Neutral red uptake of Caco-2 after 24 h exposure to different methanolic extracts: (a) leaves from* C. ambrosioides *(500 *μ*g/ml ELM); (b) fruits from* C. ambrosioides *(50 *μ*g/ml ESM); (c) bark of* P. dulce* (400 *μ*g/ml GBM); (d) leaves from* P. dulce* (100 *μ*g/ml GLM).

Even though 500 *μ*g/ml of ELM reduced the confluence of Caco-2 strongly, the cells were disintegrated and the lysosomes were not stained by NRU (Figure 4(a)), while at 250 *μ*g/ml of ELM only the confluence was reduced but the lysosomes were still functional (not shown). In contrast, the extract from its fruits (ESM) at 20 *μ*g/ml reduced slightly confluence; at 50 *μ*g/ml of ESM confluence was clearly reduced and the cells were deformed and barely stained by NRU (Figure 4(b)) and many cells were stained blue by TBS (not shown). At higher concentrations (≥100 *μ*g/ml ESM) only cell debris could be observed (not shown; similar to the positive control Figure 3(f)). This cytotoxic effect was already seen after 4 h of exposure (not shown). GBM at 400 *μ*g/ml diminished confluence and the cells were not stained red by NRU (Figure 4(c)); GLM at 100 *μ*g/ml diminished lysosomal functionality only slightly (Figure 4(d)) and cells were not stained blue by TBS (not shown), but already at 200 *μ*g/ml of GLM the cell membrane was disrupted (not shown) and lysosomes were not functional anymore (not shown).

## 4. Discussion

The methanolic extracts were evaluated against ten bacterial strains; three of them were Gram-positive and seven Gram-negative and all of them are of clinical interest. The cell line used was Caco-2 (human epithelial colorectal adenocarcinoma) in order to elucidate possible antitumoral effects.

*Amphipterygium adstringens*. The methanolic raw extract of the bark of* A. adstringens* (CBM) was cytotoxic for the cell line Caco-2 at a concentration of CC_50_ = 342 ± 38 *μ*g/ml, which is in a similar range of observed cytotoxic effects of an aqueous (CBW) and an ethanolic bark (CBE) extract on HeLa with a CC_50_ = 467 *μ*g/ml and CC_50_ = 202 *μ*g/ml, respectively [38]. It was previously shown that the bark contains anacardic acids [39], and those compounds were cytotoxic to several cell lines at concentrations of 10–40 *μ*M (≈3.4–13.6 *μ*g/ml) [27, 40]; as the cytotoxicity increased with decreasing polarity of the solvent, it is reasonable that those lipophilic anacardic acids are the cytotoxic principle for the cell line Caco-2 as well. Also Rodriguez-Garcia et al. (2015) found antiproliferative effects by a methanolic bark extract (CBM) on human cell lines like OVCAR-3, UACC-62, HT-29, PC-3, U251, NCI-H460, and 786-O ranging from 4.4 to 28.0 *μ*g/ml [41]. On the other hand, Xiu et al. (2014) showed that anacardic acids promote proliferation of ovarian cancer cells, inhibit late apoptosis, and induce cell migration by lamellipodia formation [42]. Surprisingly, our CBM extract did not inhibit bacterial growth, although we found that an ethanolic extract (CBE) inhibited the growth of* B. cereus* (150 *μ*g/ml),* E. faecalis* (660 *μ*g/ml), and* S. aureus* (1,380 *μ*g/ml) [38]. Canales et al. (2005) reported that an methanolic extract inhibited* Sarcina lutea* (125 *μ*g/ml),* S. aureus* (250 *μ*g/ml), and* B. subtilis* (1,500 *μ*g/ml) [43], although Rodriguez-Garcia et al. (2015) reported only an effective inhibition of* Streptococcus mutans* (125 *μ*g/ml CBM) while other bacteria are only inhibited at very high concentrations (>37 mg/ml CBM) [41]. This apparent contradiction may be explained by the fact that the degree of unsaturation of the fatty acid of anacardic acids has a high impact on their antimicrobial activity [44].

*Cirsium mexicanum*. To the best of our knowledge, there are no scientific reports on the biological activity of extracts from* C. mexicanum*, so we report here for the first time that extracts from the whole plant as well as from the flowers of this species contain potentially active molecules, mainly tannins and saponins, and, in contrast to the whole plant, the flowers contained also terpenoids; flavonoids were barely detectable in both extracts. Using the Kirby-Bauer test, no antibacterial activity could be detected and low cytotoxicity was observed for both methanolic extracts with 323 ± 93 *μ*g/ml CsM and 250 ± 55 *μ*g/ml CsFM, respectively. The latter results could be confirmed qualitatively by NRU and TBS. These results do not indicate this plant as a good candidate for anticancer treatment as it is used empirically by the Mexican population [14].

*Eryngium carlinae*. On the composition and biological activity of extracts from* E. carlinae* few scientific reports exist only: for the aerial parts of some* Eryngium* species it is known that they contain mainly saponins, flavonoids, and essential oils [45]; here we report for the first time a qualitative phytochemical analysis for the species* E. carlinae*: saponins and tannins were abundant; flavonoids and terpenoids were hardly detectable. While Castillo-Juárez et al. (2009) reported that a methanolic extract inhibited* H. pylori* [16], we could not find bacterial inhibition using the Kirby-Bauer method. A similar result was found for methanolic extracts from leaves of* E. foetidum*, which is also native to Mexico and South America: one group reported inhibition of* H. pylori*, while another group found no inhibition of several other bacterial strains (reviewed in [45]). To our knowledge, here we report for the first time cytotoxicological results on* E. carlinae*: we found with 356 ± 114 *μ*g/ml HsM low cytotoxic effects on Caco-2. Until now, only for few triterpenoids and triterpenoid-glycosides, isolated from different* Eryngium* species, there are reports about their moderate antiproliferative activity on different human cell lines [45].

*Pithecellobium dulce*. Kumar and Nehra (2014) found antibacterial activity for a methanolic bark extract from* P. dulce* against* E. faecalis*,* E. coli*,* K. pneumoniae*,* M. luteus*,* S. typhimurium*, and* S. epidermidis* at MICs of 250–500 *μ*g/ml [46] and the same group reported inhibition of bacterial growth for a methanolic leaf extract against* B. subtilis*,* E. aerogenes*,* E. coli*,* K pneumoniae*,* P. aeruginosa,* and* S. epidermidis* at MICs ranging from 375 to 1000 *μ*g/ml [47]. We were not able to detect bacterial inhibition using the Kirby-Bauer test, from both extracts, but we found a cytotoxic effect on Caco-2 with CC_50_ = 347 ± 78 *μ*g/ml for the bark extract (GBM) and the methanolic leaf extract (GLM) was even more cytotoxic with a CC_50_ = 126 ± 14 *μ*g/ml. This is in a similar range of CC_50_ = 112 *μ*g/ml GLM for the cell line MCF-7 [48], a value also reported by Olmedo-Agudo et al. (2016) with CC_50_ > 100 *μ*g/ml GLM [21]; the latter group determined cytotoxicity for the cell line CaP with CC_50_ = 3.7 *μ*g/ml, indicating that* P. dulce* leaf extract has cytotoxic potential.

*Chenopodium ambrosioides*. The leaves of* C. ambrosioides* especially have been broadly studied, but the results vary strongly. While some authors find ascaridole and derivates with about 50–80.0% as the main component [33, 49–51], others find it with less than 10% as a minor component [52, 53]. It is supposed that, apart from the different geographic location, the part of the plant, or the processing method, those differences can be explained by the existence of different varieties of the plant:* C. ambrosioides* var.* anthelminticum* has a high ascaridole content while the other variety, var.* pubescens*, does not [54]. Sousa et al. (2012) could not detect antimicrobial activity from an ethanolic extract of the aerial parts up to 500 *μ*g/ml [55], as we did not observe it either for a methanolic extract from the leaves. Jesus et al. (2017) determined for an ethanolic crude extract from leaves only very high MICs against* Mycobacterium tuberculosis* (1.25 mg/ml),* E. faecalis* (4.29 mg/ml), and* P. aeruginosa* (68.75 mg/ml) [56]. Generally, the whole aerial part is not considered (very) cytotoxic: Ruffa et al. (2002) did not detect cytotoxic effects up to 1,000 *μ*g/ml for HepG2 [57], Koba et al. (2009) found a CC_50_ = 700 *μ*g/ml for HaCaT [50], and Barros et al. (2013) determined CC_50_ of 264 to 319 *μ*g/ml for HeLa, HepG2, and HCT-15 [58]; those values are in the range of CC_50_ = 563 ± 66 *μ*g/ml ELM that we report for Caco-2. Although Degenhardt et al. (2016) reported cytotoxic effects of an ethanolic leaf extract against blood cell lines with CC_50_ of 30–62 *μ*g/ml [33] and Al-kaf et al. (2016) reported a CC_50_ ≈ 25 *μ*g/ml for HT-29 for a hydrodistillate [51], both associated the cytotoxic effect to the high ascaridole content of their extracts, which coincide with a report on the cytotoxicity of ascaridoles with CC_50_ = 4.2–23.7 *μ*g/ml for different tumour cell lines [59]. However, Koba et al. (2009) could demonstrate that it is neither ascaridole (CC_50_ > 1 mg/ml), as suspected, nor* p*-cymene (CC_50_ > 1 mg/ml), but neral (CC_50_ ≈ 100 *μ*g/ml) is the cytotoxic principle [50]. Mainly we have analysed the parts of the plants commonly used to treat several disorders, including cancer, according to indigenous healers from Mexico; the only exception being the leaves from* C. ambrosioides*, which are preferred over their fruits. Here we report for the first time the cytotoxic properties of the methanolic fruit extract, being highly cytotoxic with a CC_50_ = 45 ± 7 *μ*g/ml, killing the cells by necrosis within about 4 h. Moreover, we found also antibacterial properties of this extract being especially effective against* S. typhimurium* with a MIC of 137 *μ*g/ml ESM. Only Ajaib et al. (2016) have reported inhibition zones of 20–30 mm in agar-diffusion tests against* E. coli*,* S. aureus*,* P. aeruginosa,* or* B. subtilis* by methanolic extracts from the fruits [32]. This makes the fruit extract of* C. ambrosioides* an interesting candidate for further studies on biological activity. A preliminary qualitative phytochemical analysis revealed the presence of flavonoids, tannins, and terpenoids and the absence of saponins.

## 5. Conclusion

This ethnopharmacological evaluation helps to direct the screening for plants with antitumoral properties. The most promising candidate resulted to be the methanolic extract of fruits from* C. ambrosioides* (ESM), followed by the methanolic leaf extract from* P. dulce* (GLM). In further studies the cytotoxic and antibacterial principle, especially from ESM, will be elucidated.

## Figures and Tables

**Table 1:** Fractions of the plants employed and concentration of the methanolic extracts. Previously reported biological activity of plant extract and their reference.

Plant sample	Plant fraction		Action	Reference
Voucher				
*Amphipterygium adstringens* (cuachalalate) CBL201701	Bark CBM 80 mg/ml		Antibacterial	[10, 24–26]
			Cytotoxic	[24, 27]
			Proliferation	[2]

*Chenopodium ambrosioides* (epazote) CBL201702	Leaf ELM 20 mg/ml	Fruit ESM 20 mg/ml	Antiprotozoal	[25, 28–30]
			Antifungal	[31]
			Antibacterial	[32]
			Cytotoxic	[33]
			Not cytotoxic	[9]

*Cirsium mexicanum* (cardosanto) CBL201703	Flower CsFM 48 mg/ml	Aerial parts CsM 40 mg/ml	Anticancer	[14]
			Antidiabetic	[14]
	

*Eryngium carlinae* (hierba del sapo) CBL201704	Aerial parts HsM 50 mg/ml		Antidiabetic	[30]
		
		

*Pithecellobium dulce* (guamúchil) CBL201705	Bark GBM 20 mg/ml	Leaf GLM 20 mg/ml	Antibacterial	[34]
			Cytotoxic	[1]
			Not cytotoxic	[1]

**Table 2:** Qualitative phytochemical analysis of 4 studied extracts: ++, present; +, slightly present; and −, not present.

Extract	Flavonoids	Tannins	Terpenoids	Saponins
ESM	++	++	++	−
CsM	+	++	+	++
CsFM	+	++	++	++
HsM	+	++	+	++

**Table 3:** Molecular identification of the collected medicinal plants through DNA barcoding.

Plant sample	DNA barcode	GenBank ID	Top scores	% similarity
*Chenopodium ambrosioides (Dysphania ambrosioides)*	*rbcL*	MG436873	*D. ambrosioides*	100.00
			*D. multifida*	100.00
			*C. album*	99.62
	*matK*	MG572013	*D. ambrosioides*	100.00
			*C. acuminatum*	99.88
			*D. multifida*	99.74

*Cirsium mexicanum*	*rbcL*	MG436872	*C. mexicanum*	100.00
			*C. undulatum*	100.00
			*C. scariosum*	100.00
	*matK*	MG572012	*C. discolor*	100.00
			*C. altissimum*	100.00
			*C. horridulum*	100.00

*Eryngium carlinae*	*rbcL*	MG436874	*E. aristulatum*	99.81
			*E. pendletonense*	99.81
			*E. leavenworthii*	99.62
	*matK*	No data	No data	No data

*Pithecellobium dulce*	*rbcL*	MG436875	*P. dulce*	100.00
			*P. lanceolatum*	99.43
			*Acacia centralis*	99.43
	*matK*	MG572014	*P. dulce*	100.00
			*P. lanceolatum*	100.00
			*Havardia mexicana*	99.25

**Table 4:** Antibacterial activity of methanolic extract from *Chenopodium ambrosioides* fruits (ESM) compared to chloramphenicol (Cm). Values expressed as mean ± 1.96 *∗* SEM (*n* = 3) of the inhibition halo in mm.

Microorganism	Zone of growth inhibition [mm]		Statistical
	ESM (10 *μ*l, 70 mg/ml)	Cm (2 *μ*l, 50 mg/ml)	significance
*Enterococcus faecalis*	13.3 ± 2.4	22.0 ± 1.1	*p* = 0.003
*Salmonella typhimurium*	18.0 ± 1.1	37.3 ± 3.5	*p* = 4.8 *∗* 10^−4^
*Escherichia coli*	12.0 ± 0.6	26.3 ± 2.4	*p* = 3.2 *∗* 10^−4^

**Table 5:** Minimal inhibitory concentration (MIC) for methanolic extract from *Chenopodium ambrosioides* fruits (ESM) compared to chloramphenicol (Cm).

Microorganisms	Minimal inhibitory concentration (MIC) [*µ*g/ml]	
	ESM	Cm
*Enterococcus faecalis*	4375	6
*Salmonella typhimurium*	137	6
*Escherichia coli*	1094	6

## Abbreviations

MTP: Microtiter plate
NRU: Neutral red uptake
TBS: Trypan blue staining
CBM: Cuachalalate bark methanolic extract
CsFM: Cardosanto flower methanolic extract
CsM: Cardosanto aerial parts methanolic extract
ELM: Epazote leaf methanolic extract
ESM: Epazote fruit (seed) methanolic extract
GBM: Guamúchil bark methanolic extract
GLM: Guamúchil leaf methanolic extract
HsM: Hierba del sapo aerial parts methanolic extract.
